# Gender and sex differences in colorectal cancer screening, diagnosis and treatment

**DOI:** 10.1007/s12094-024-03801-0

**Published:** 2025-01-17

**Authors:** Encarnación González-Flores, Rocio Garcia-Carbonero, Elena Élez, Eduardo Redondo-Cerezo, María José Safont, Ruth Vera García

**Affiliations:** 1 Department of Medical Oncology, Hospital Universitario Virgen de las Nieves, Av. de las Fuerzas Armadas, 2, Beiro, 18014 Granada, Spain; 2 Instituto de Investigación biosanitaria.ibs.granada, Granada, Spain; 3 Department of Medical Oncology, Hospital Universitario 12 de Octubre, Imas12, Medicine Faculty, Universidad Complutense Madrid (UCM), Madrid, Spain; 4 Department of Medical Oncology, Vall d’Hebron Hospital Campus and Institute of Oncology (VHIO), Universitat Autònoma de Barcelona, Barcelona, Spain; 5 Department of Gastroenterology, Hospital Universitario Virgen de las Nieves, Granada, Spain; 6 Department of Medicine, The University of Granada, Granada, Spain; 7 Department of Medical Oncology, University General Hospital of Valencia, Valencia University, CIBERONC, Valencia, Spain; 8 Department of Medical Oncology, University Hospital of Navarra, Instituto de Investigación Sanitaria de Navarra, IdISNA, Navarra, Spain

**Keywords:** Colorectal cancer, Diagnosis, Gender, Screening, Sex, Treatment

## Abstract

Males have a higher incidence and mortality rate from colorectal cancer (CRC) compared with females. This review examines the reasons for these differences, including risk factors, screening participation, interpretation of screening tests, presentation and tumour types, pathophysiology (particularly the impact of sex hormones on tumour-related gene expression, microsatellite instability, micro-RNA expression, and the tumour microenvironment), and the efficacy and toxicity of treatment. Sex differences in hormones and body composition are responsible for some of the sexual dimorphism in CRC incidence and outcomes, particularly the pathophysiology, CRC presentation, the pharmacokinetics of cytotoxic therapies, and the impact of treatment on outcomes. However, gender differences also play a role, affecting risk factors, access to or participation in screening and treatment, and patients’ experience of treatment (e.g. adverse events and sequelae). Sex and gender issues warrant further investigation in CRC to optimise treatment outcomes for patients.

## Introduction

Colorectal cancer (CRC) is one of the most common forms of cancer in the world—the second most prevalent in women (after breast cancer) and the third most prevalent in men (after prostate and lung cancer) [1]. However, the age-standardised incidence and mortality rates differ between the sexes, with men having a higher rate of diagnosis (23.4 vs 16.2 per 100,000) and death (11 vs 7.2 per 100,000) compared with women [1]. Many factors contribute to these differences in epidemiology and outcomes between men and women, including factors related to sex and gender [2].

‘Sex’ and ‘gender’ are not interchangeable terms. Sex refers to the genetically determined biological difference based on chromosomes, reproductive organs, hormones and body composition [3]. Gender refers to the social construct that defines the societal norms, roles and behaviours expected of the different sexes [4]. Both sex and gender affect outcomes for patients with CRC via biological mechanisms and differences in lifestyle, health behaviours and access to medical care [2].

The Sociedad Española de Oncología Médica (SEOM) has developed a Task Force to raise awareness about sex and gender issues in cancer care in Spain [5]. The aim of the current narrative review is to examine the impact of sex and gender on outcomes in patients with CRC.

## Materials and methods

A search of PubMed was conducted in January 2024, first using terms for (“sex” OR “gender”) AND (“colon cancer” OR “colorectal cancer”), and then combining the results of this search with specific terms relating to epidemiology (including frequency, incidence and prevalence), pathogenesis and treatment. The articles identified were evaluated for relevance and were supplemented by additional articles identified from the bibliographies of identified articles or ad hoc searches in relation to specific topics.

## Epidemiology of CRC

### Incidence/risk

**Fig. 1:**
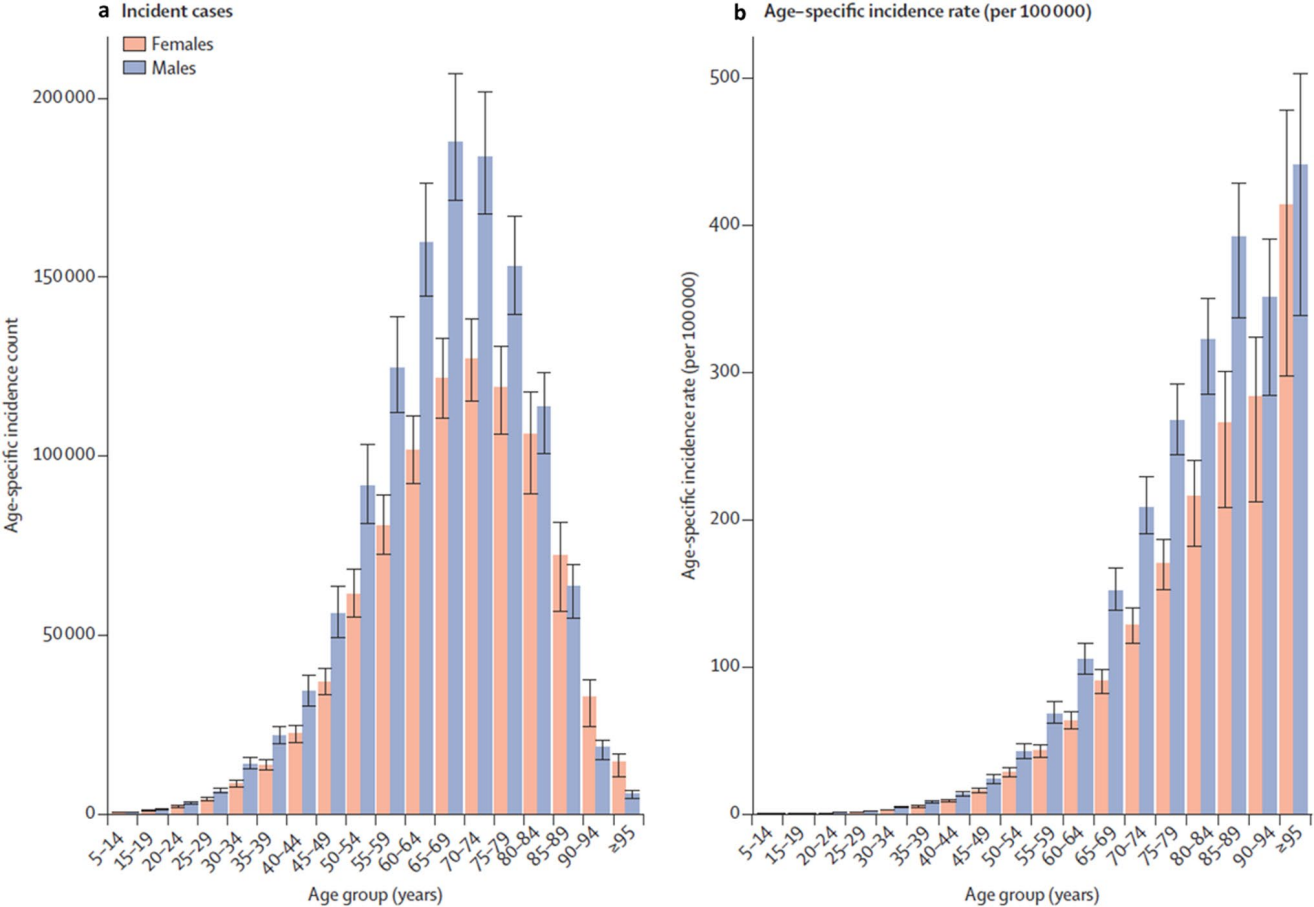
The global incidence of colorectal cancer in 2019, stratified by age at diagnosis, and shown as **a** number of incident cases, and **b** age-specific incidence [6]. Reproduced from Fig. 5 in [6], under a CC BY 4.0 DEED license (https://creativecommons.org/licenses/by/4.0/)

Across most age groups, the incidence of CRC is higher in men than women, but the difference is most marked between the ages of 55 and 84 years (Fig. 1); in people aged ≤ 54 years and ≥ 85 years, the number of incident cases is generally similar in the two sexes or slightly higher in females [6]. Similar to global statistics, the age-standardised incidence of CRC in Spain in 2020 was higher in males than females (25.6 vs 17.5 per 100,000) [1]. Potential reasons for these differences may be related to risk factors specific to sex or gender.

#### Sex

One potential reason for the sex differences in CRC incidence is the body composition of males versus females. Obesity is a known risk factor for CRC in both sexes, and body mass index (BMI) is a risk factor for CRC in men at any age [7]. However, in early life (up age 21 years), BMI is a stronger risk factor for CRC in women than in men [7]. The Nurses’ Health Study II showed that BMI at age 18 years and weight gain after age 18 years were both risk factors for early-onset CRC (before age 50 years) in women; each 5-unit increase in BMI was associated with a 20% increase in the risk of developing CRC (hazard ratio [HR] 1.20; 95% confidence interval [CI] 1.05–1.38; *p* = 0.01), and each 5 kg of weight gain after age 18 years was associated with a 9% increase (HR 1.09; 95% CI 1.02–1.16; *p* = 0.007) [8]. Abdominal adiposity may be key in mediating the risk of CRC, because, in adult women, waist-to-hip ratio is a stronger predictor of CRC risk than is BMI [9].

The sex differences in CRC incidence may also be hormone related, but research on the relationship between circulating levels of endogenous sex hormone and CRC risk have produced mixed results. Endogenous levels of testosterone and sex hormone-binding globulin (SHBG) were inversely associated with the risk of developing CRC in men [10]; similarly there seems to be an inverse relationship between endogenous oestradiol or SHBG levels and CRC risk in postmenopausal women [11]. However, a meta-analysis of available data did not find any relationship between endogenous sex hormone levels and CRC risk in men or postmenopausal women [12].

Endogenous oestrogen protects against microsatellite instability (MSI) in younger women, but women develop MSI+ tumours at a high rate after menopause, and hormone replacement therapy (HRT) is protective against this [13]. Sex hormones are likely to mediate the effect of adiposity on cancer risk [7, 14], since adipose tissue is a key source of oestrogen production in men and postmenopausal women and leads to an increase in the oestrogen/testosterone (E/T) ratio [7]. After adjustment for BMI, a high E/T ratio was associated with a significantly increased risk of CRC in men, but a significantly reduced risk in women [10]. This suggests that the CRC risk increases in men when there is a loss of testosterone and increase in oestrogen (which will increase the E/T ratio) and in women when there is an increase in testosterone and reduction in oestrogen (which will reduce the E/T).

Supporting a potentially protective effect of oestrogen are data showing that exogenous oestrogen appears to protect against CRC development, such that women who have used hormonal contraceptives in their lifetime or HRT after menopause are at a reduced risk of CRC development [15, 16].

#### Gender

The difference in CRC incidence may be at least partially explained by differences in lifestyle and health behaviours between men and women. Men tend to eat more meat and drink more alcohol than do women, and are more likely to be heavy smokers and to have a sedentary lifestyle [17]. Moreover, the effect of a pro-inflammatory lifestyle (i.e. a diet with a low Inflammatory Score of the Diet [ISD], the presence of abdominal obesity and low levels of physical activity) on colon cancer risk is greater in men than in women [18]. Data suggest that 22% of the CRC risk in European males and 11% in European females can be attributed to not adhering to healthy lifestyle factors or behaviours (based on bodyweight, physical activity, alcohol consumption, smoking and a healthy diet) [19]. Data from Spain show that eating a mostly Western diet (rich in high-fat dairy products, processed meat, refined grains, etc. and poor in low-fat dairy products, fruits and vegetables) significantly increases the risk of CRC in Spanish women, but not in men, whereas adherence to a Mediterranean diet (high intake of fish, vegetables, fruit and legumes) is protective against CRC development in men but not in women [20].

### Survival

Mortality rates from CRC are generally lower in females than males [21]. In Spain, lower age-adjusted rates of CRC mortality are seen in women compared with men [22, 23], but the data are more nuanced than simple sexual dimorphism.

Data from two Spanish cancer registries found no differences in early (6- or 12-month) survival between male and female patients with CRC; the presence of multimorbidity was a more important prognostic factor than sex was in this analysis [24]. Socioeconomic status has been found to be an important determinant of survival in CRC patients, including in Spain [23]. Consistent with this finding, researchers in the United States (US) identified geographical ‘hot spots’ with high mortality from early-onset CRC amongst women, and found these areas had high numbers of Black residents, a higher incidence of obesity and unemployment, low levels of physical activity and educational attainment, and a high number of children [25]. Gender is an important determinant of socioeconomic position [26]. Gender inequality is not as marked in Spain as it is in some countries, but Spanish women are still disadvantaged in work opportunities/pay and educational outcomes relative to men, and undertake a disproportionate share of domestic, child-rearing and caregiving activities [27], all of which negatively affect the socioeconomic status and lifestyle options of women.

Stage at diagnosis is an important determinant of mortality in CRC [28]. Data from Canada indicate that psychosocial factors (such as having a life partner) and diet-related factors predicted early-stage CRC diagnosis in men, whereas health behaviours (such as obtaining regular Pap smears) predicted early CRC diagnosis in women [29], all of which are gender- rather than sex-related risk factors.

## Diagnosis

Outcomes in CRC are dependent on tumour stage at diagnosis; the 5-year survival rate when CRC is identified at a localised stage is around 90% [30]. Therefore, early diagnosis is important.

Most developed countries, including Spain, offer population-based screening for CRC to identify tumours at an early stage and therefore optimise outcomes [31]. Screening increases the likelihood of identifying CRC at stage I or II relative to diagnosis based on symptoms [32].

Screening programmes generally use faecal occult blood tests (FOBT) or the more sensitive and specific faecal immunochemical tests (FIT), although countries differ in the choice of test, the eligible age group and frequency of such tests [31]. In Spain, adults aged 50–69 years are offered FIT screening every two years [33]. Individuals with a positive test are referred for colonoscopy.

There is clear evidence for sex/gender differences in screening participation internationally [34–36]. Women tend to participate in CRC screening programmes at a higher rate than do men, including in Spain [34–37], although this is not a universal finding around the world [38]. Screening participation is also affected by sociodemographic and contextual factors that may be influenced by gender, such as income (lower participation amongst those of lower socioeconomic status), health behaviours, comorbidities, place of residence (lower participation amongst urban vs rural populations), and family support (higher participation with more family support) [34–36, 38, 39]. Qualitative research indicates that men are more likely than women to have ‘avoidant procrastination with underlying fatalism’ in relation to CRC screening (i.e. an attitude of “what you don’t know can’t hurt you”) or to consider that there is no need to engage in health interventions if no symptoms are present [39].

Data from the US suggest that removal of financial barriers increases the participation of women, but not men, in endoscopic screening programmes [40]. Participation in endoscopic screening may also be influenced by age- and gender-related preferences about the sex of the endoscopy team, especially amongst women and people aged 50–60 years [41].

**Fig. 2:**
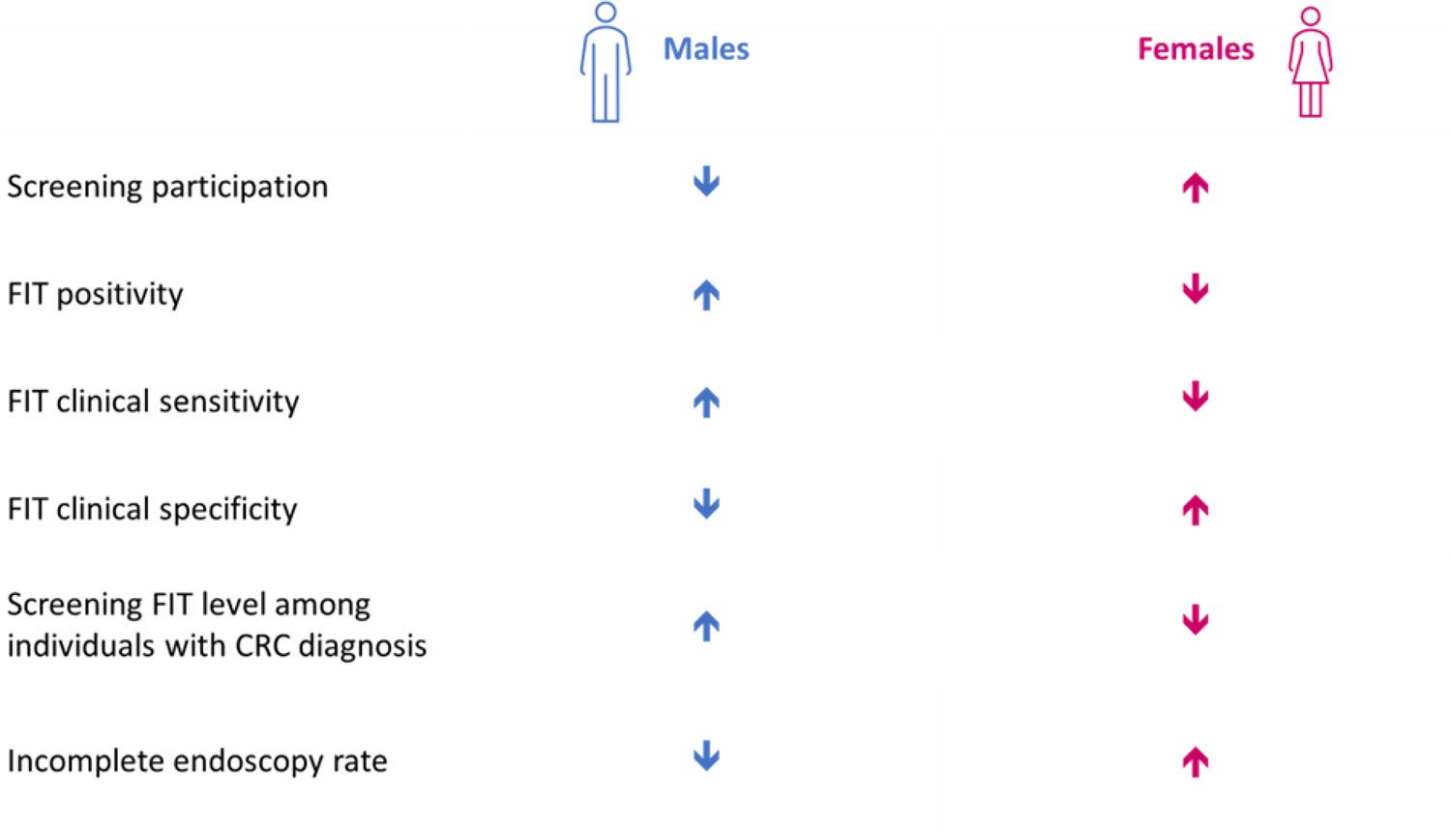
Potential reasons for the difference in CRC screening effectiveness between males and females [34]. *CRC* colorectal cancer, *FIT* faecal immunochemical test (for haemoglobin)

There are also differences between men and women in the effectiveness of screening to detect CRC (Fig. 2) [34]. CRC screening tends to have lower sensitivity in women compared with men (i.e. higher rate of false negatives), but better specificity in women than men (i.e. lower rate of false positives). Because women are less likely than men to return a positive result, they are also less likely to undergo further investigation. For example, a study in Spain reported that the population of patients referred for colonoscopy after a positive screening test comprised 61.2% males and 38.8% females [42]. However, such sex-related differences have not been consistently reported in the literature [43, 44].

One reason for the higher false-negative rate in women is that women with CRC have lower levels of haemoglobin in stool. Indeed, there is evidence to suggest that sex-specific thresholds should be applied when interpreting FIT results [45]. When the same haemoglobin threshold is applied to FIT interpretation in both sexes, women are more likely than men to have interval cancers (i.e. CRC tumours detected between screening tests) [46]; however, when a lower haemoglobin threshold is applied to interpreting FIT samples from female patients, the interval cancer detection rate is higher in men, consistent with the higher CRC incidence rate [37]. According to an analysis from the Basque region in Spain, where the threshold haemoglobin level was 20 μg/g in both sexes, the positive predictive value, positivity rate, CRC-detection rate and advanced CRC-detection rate from screening were all significantly higher in men than women [47]. The number needed to screen to detect one case of CRC was 59 in men and 92 in women at this FIT threshold [47].

Another reason for the high false-negative rate amongst women is that females are more likely than males to develop right-sided CRC [48], and right-sided disease is associated with a significantly higher rate of false-negative FIT results [49]. In addition, women are less likely than men to have adenoma detected during colonoscopy [37, 42, 50], and adenoma detection is significantly associated with a positive FIT result [50, 51]. A Finnish study reported that, in individuals who underwent colonoscopy as a result of a positive screening test, the rate of detection of adenoma was significantly lower (risk ratio [RR] 0.94; 95% CI 0.89–0.99; *p* = 0.004) and rate of mucinous adenoma detection significantly higher (RR 1.98; 95% CI 1.22–3.25; *p* = 0.004) in female than male patients [37].

On the other hand, women are more likely than men to have sessile serrated lesions detected on colonoscopy, and these lesions may not be detected by FIT because they rarely bleed [51]. Interestingly, in the Finnish study described above, women who underwent screening were significantly less likely than men to be asymptomatic (RR 0.75; 95% CI 0.60–0.93; *p* = 0.011), with women showing a significantly increased rate of abdominal pain (RR 1.65; 95% CI 1.20–2.26; *p* = 0.002) [37]. This suggests that asymptomatic men may derive the greatest benefit from screening, consistent with the Spanish data showing a lower number needed to screen in men than in women [47].

## Anatomical and molecular differences

**Fig. 3:**
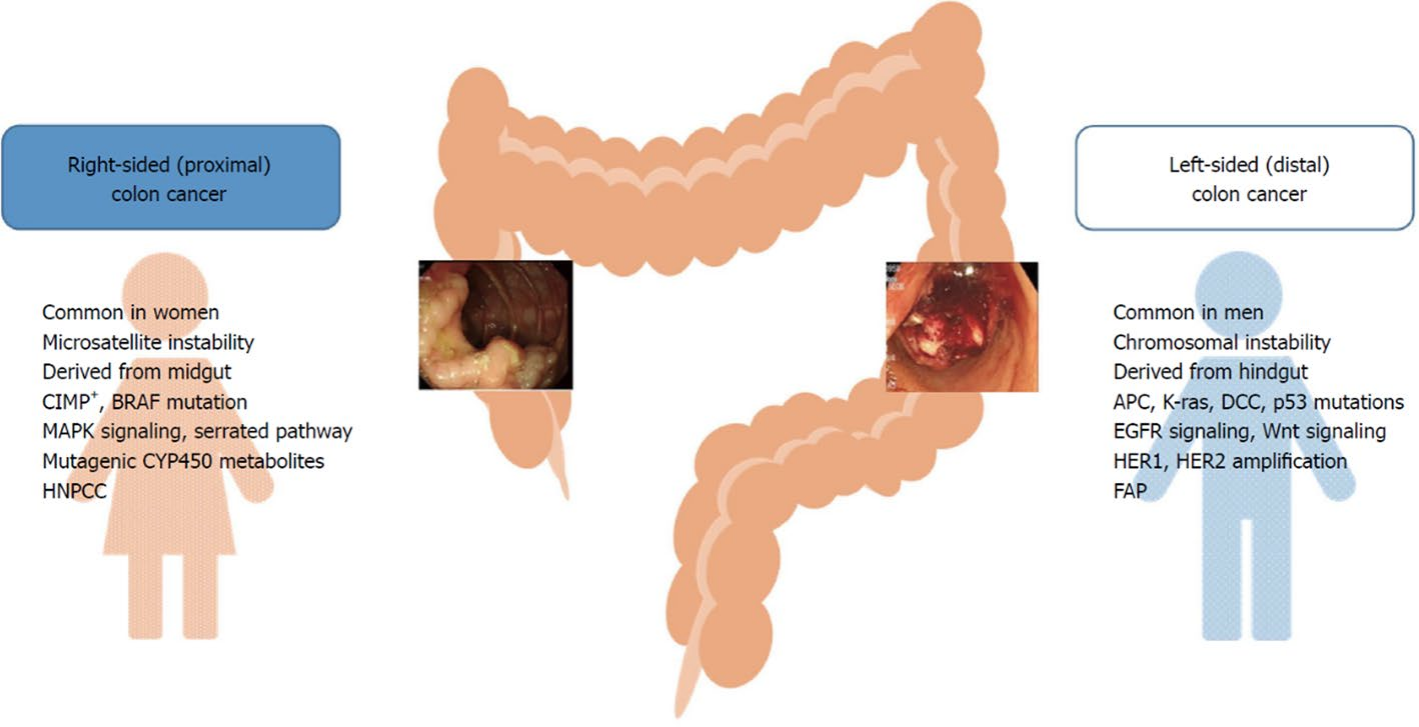
Clinical and molecular characteristics of left- and right-sided tumours of the colon [48] *APC* adenomatous polyposis coli, *BRAF* v-raf murine sarcoma viral oncogene homolog B1, *CIMP* CpG island methylator phenotype, *CYP450* cytochrome P450, *DCC* deleted in colorectal cancer, *EGFR* endothelial growth factor receptor, *FAP* familial adenomatous polyposis, *HER* human epidermal growth factor receptor, *HNPCC* hereditary non-polyposis colorectal cancer, *K-ras* Kirsten ras, *MAPK* mitogen-activated protein kinase. Reproduced from Fig. 1 in 48, under a CC BY 4.0 DEED license (https://creativecommons.org/licenses/by/4.0/)

As described above, females are more likely to develop proximal (right-sided) CRC whereas males are more likely to develop distal (left-sided) CRC and adenomas [48, 50], and the clinical and molecular characteristics differ between these sites (Fig. 3). Differences in the cellular and molecular characteristics can lead to differences in tumour behaviour and prognosis, with right-sided tumours showing a more aggressive phenotype that is less responsive to chemotherapy [52].

Hormones are thought to play a key role in the sexual dimorphism of CRC development, with oestrogen and the membrane-bound G protein-coupled oestrogen receptor (GPER) regulating key physiological functions in the intestine, including oestrogen receptor (ER) β expression and the actions of specific potassium channels (KCNQ1:KCNE3), which control transepithelial electrolyte transport in the colon and act as tumour suppressors [53]. Differential expression of ERβ between males and females may explain some of the epidemiological differences in CRC rates, particularly the protective effect of oestrogen [52].

Data indicate that the tumour microenvironment (TME) also differs between men and women, with women showing higher levels of CD4+ lymphocytes in tumour tissue, lymph nodes and uninvolved colon tissue compared with men; CD8+ levels in tumour and lymph nodes were similar in the two sexes, but women showed greater CD8+ infiltration in uninvolved peri-tumoural tissue [54]. Tumours from females with CRC also show different expression of genes involved in regulatory T-cell (Treg) function (higher *GOT1* and *GHR* expression, lower *DAB2*, *TNFRSF25* and *LRRC32* expression), T helper 1 (Th1) response (higher expression of *IL18R1, GBP1* and *STAT4*), co-stimulatory T-cell markers (higher expression of *CD96, DPP4, GZMK* and *CCL14*), and CD8+ cell exhaustion (higher expression of *PD-L1* and *TIGIT*) compared with male tumours [54]. According to data from The Cancer Genome Atlas Program (TCGA), 57 of the 65 genes (87.7%) that are differentially expressed between males and females with CRC are located on the X or Y chromosomes [54].

Another factor that may account for the clinical and molecular differences between CRC in males and females is micro-RNA expression, with tumours from men showing significantly higher expression of miR-21-5p, miR-21-3-p and miR-16-3p compared with female tumours [55].

Whilst most of these differences are clearly sex-related, there may also be gender-related differences induced by epigenetic changes in response to environmental or behavioural factors. For example, detectable levels of p16INKα methylation, an epigenetic marker of aberrant DNA silencing, in CRC are significantly more likely to be found in women than men, in patients with proximal rather than distal tumours, and in those with poorly versus well differentiated tumours [56]. Sex differences have also been noted in how the gut microbiome responds after CRC development, with men showing a more stable microbiome and women showing a reduction in the diversity of species present [57]; the extent to which these changes are determined by sex, gender or both is not clear.

## Influence of sex and gender on the use and efficacy of treatments for CRC

Treatment decisions in CRC are highly influenced by the stage and site of the tumour, and the physical condition of the patient; therefore, treatment patterns differ by sex because women are more likely to present with right-sided disease, a more advanced stage of cancer, lymph node metastasis, and older age [58–61].

European Society for Medical Oncology (ESMO) guidelines recommend surgery for localised CRC, followed by adjuvant chemotherapy with fluoropyrimidine/oxaliplatin regimens (leucovorin + 5-fluorouracil + oxaliplatin [FOLFOX] or capecitabine + oxaliplatin [CAPOX]) for stage III or high-risk stage II tumours [62]. For most patients with unresectable stage IV CRC, chemotherapy is recommended, with the choice of treatment guided by patient status and the molecular characteristics of the tumour [63].

Amongst patients with rectal cancer, differences in pelvic anatomy between men and women can affect surgical parameters, with sex and pubic diameter being significant predictors of operative time in one Spanish analysis [64]. In the same study, previous surgery was a significant predictor of operative difficulty and the need to convert from laparoscopic to open technique in women but not in men [64].

A study from Spain in 2005 showed that men with CRC were significantly more likely to be hospitalised than women after surgery for colorectal cancer, even after controlling for age and comorbidity [65]. Similarly, data from Germany showed that men incur higher treatment costs than do women, during the early and late phases of CRC treatment, mainly driven by greater hospitalisation costs in early CRC and greater use of targeted therapies in late disease [58]. These differences may reflect some gender bias in the selection of patients for hospital admission/discharge or treatment, or they may reflect inequity in access to costly therapies.

Other studies also show that, in the older age groups, women with advanced CRC are less likely than men to receive systemic treatments (including adjuvant or neoadjuvant therapy) and more likely to receive best supportive care or to die without receiving treatment [61, 66, 67]. It appears that women are also less likely than men to receive any radiotherapy even when it is indicated, or adequate radiotherapy when it is administered [68].

Fluoropyrimidine-based chemotherapy is widely used for CRC, but the pharmacokinetics of these agents are significantly influenced by sex, with lower elimination and therefore greater systemic exposure in females than males [69, 70]. Because of the greater systemic exposure, rates of adverse events (AEs) are higher in female than male patients receiving these agents [2, 71, 72]. A meta-analysis of studies with 5-fluorouracil-based chemotherapy for metastatic CRC found no difference in overall survival (OS) or progression-free survival (PFS) between male and female patients, but significantly higher rates of many AEs (alopecia, diarrhoea, nausea and vomiting, anaemia and neutropenia) in female than male patients [73]. The pharmacokinetic differences between the sexes can be attributed to differences in body composition, making an argument for fluoropyrimidine dosing based on fat-free mass rather than body surface area or weight [69].

**Fig. 4:**
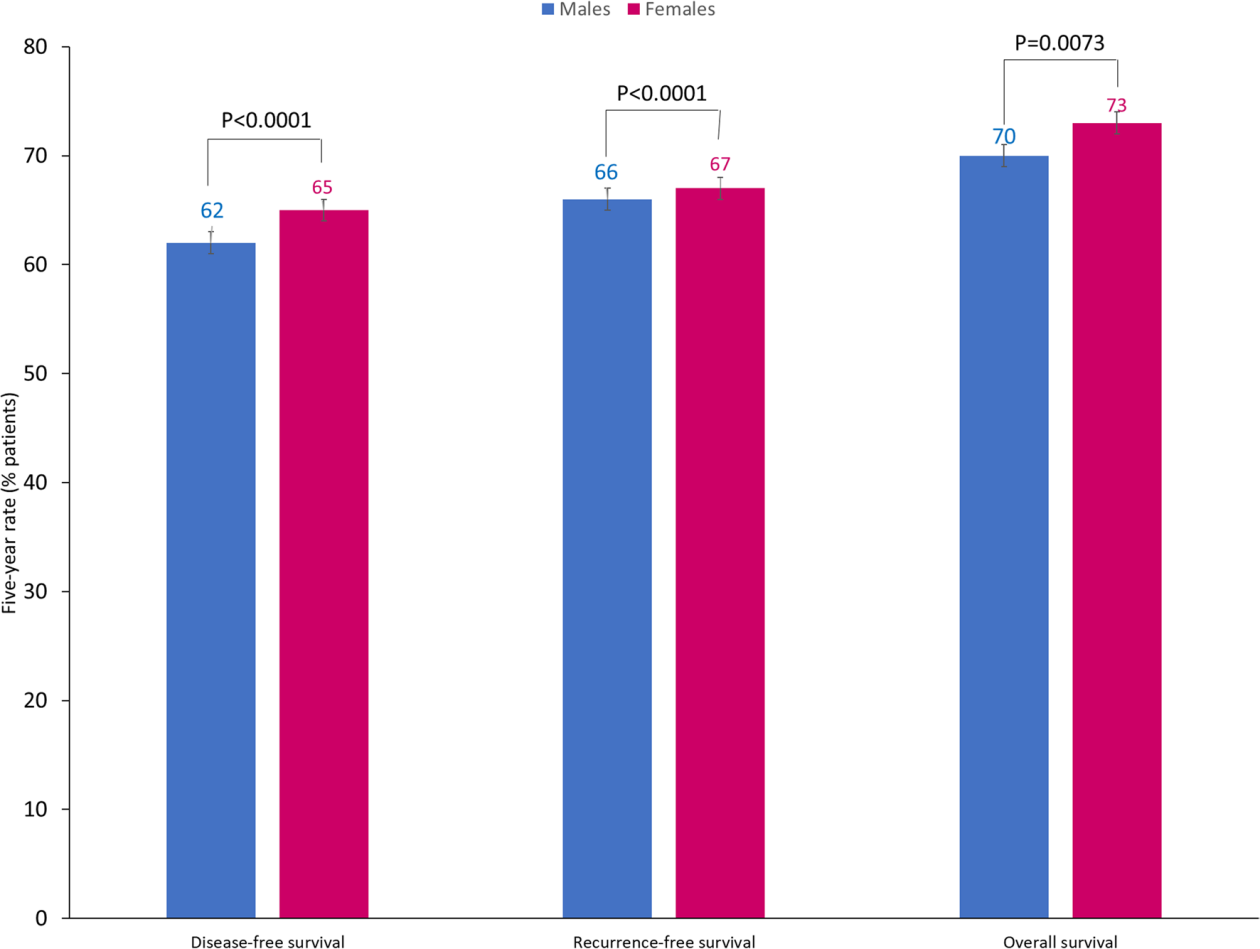
Five-year rates (95% confidence intervals) of disease-free survival, recurrence-free survival and overall survival in male (*n* = 18,224) and female (*n* = 15,101) patients with early-stage colon cancer receiving fluoropyrimidine-based chemotherapy: data from the ACCENT database [74]

The impact of sex on the efficacy of adjuvant chemotherapy is unclear. The ACCENT database of 33,345 patients with colon cancer reported that sex was not a significant predictor of treatment efficacy, but compared with males, females had slightly better 5-year rates of disease-free survival, OS and recurrence-free survival, that were statistically significant (Fig. 4) [74]. However, in the MOSAIC study, the difference between adjuvant FOLFOX and adjuvant leucovorin + 5-fluorouracil was significantly more marked in males than females, and male sex was a prognostic predictor of better OS outcomes [75].

With regard to systemic therapies for metastatic CRC, there does not appear to be a difference in outcomes between men and women receiving bevacizumab in combination with FOLFOX chemotherapy [76, 77], although a meta-analysis has suggested that, in females, the benefit of adding bevacizumab to chemotherapy is limited to patients aged ≥ 60 years, whereas men of all ages achieved a significant OS benefit from the addition of bevacizumab [76].

In the TRIBE studies, sex had no impact on objective response rate or PFS in patients randomised to FOLFOX + irinotecan (FOLFOXIRI) + bevacizumab or doublet chemotherapy + bevacizumab [78]. On the other hand, in the XELAVIRI study, men had a greater depth of response and early tumour shrinkage (ETS) when they received FOLFOXIRI + bevacizumab compared with doublet chemotherapy + bevacizumab, whereas in women, both treatment approaches were similarly effective [79]. As a result of the greater treatment response to triplet therapy in men, the overall depth of response and ETS rate were significantly higher in men than in women (both *p* < 0.0001), and ETS was associated with a significant survival benefit, even after adjustment for potential confounders [79].

In the phase 2 VALENTINO study, outcomes were similar in males and females who received panitumumab in addition to FOLFOX as first-line induction treatment in patients with *RAS* wild-type metastatic CRC [80]. However, in the PanaMa study, the effect on PFS of adding panitumumab to 5-fluorouracil + leucovorin as maintenance treatment was significant in males (HR 0.63; 95% CI 0.45–0.88; *p* = 0.006) but not in females (HR 0.85; 95% CI 0.53–1.35; *p* = 0.491), although the effect of panitumumab on OS was generally similar or slightly better in females than males [81]. Similar findings were reported in the phase 3 PARADIGM study, which found that survival outcomes were better in patients who received panitumumab compared with bevacizumab in addition to FOLFOX chemotherapy as first-line therapy for metastatic CRC, particularly *BRAF* wild-type left-sided CRC [82]. However, when stratified by sex, only males derived a significant benefit from panitumumab in OS (HR 0.77; 95% CI 0.63–0.93).

## Safety

### Toxicity of locoregional treatments (surgery and radiotherapy)

There are limited data on sex or gender differences in adverse outcomes after surgery for CRC. The CAO/ARO/AIO-94 and CAO/ARO/AIO-04 studies suggested that women had fewer postoperative complications than men after sphincter-saving surgery for rectal cancer [83]. Similarly, data from Saudi Arabia reported a significantly higher rate of surgical site infections amongst male than female patients undergoing potentially curative surgery for CRC [84]. In this study, multivariate regression analysis found that BMI was significantly associated with wound infections in men [odds ratio (OR) 1.42; 95% CI 1.13–1.78; *p* = 0.002], but the factors significantly associated with surgical site infections in women were neutrophil:lymphocyte ratio > 5 (OR 2.92; 95% CI 1.09–7.81; p = 0.033) and Glasgow Prognostic Score on postoperative day 4 (OR 2.22; 95% CI 1.47–3.34; *p* < 0.001) [84].

One study suggests that females have a more marked cortisol response to CRC surgery, even after controlling for surgical factors such as operative time and open versus laparoscopic approach [85]. A Japanese study demonstrated that health-related quality of life (HRQoL) deteriorated in the month after sphincter-saving surgery in male and female patients with rectal cancer, but improved thereafter and returned to baseline after 12 months [86]. Men experienced significantly worse social functioning, sexual functioning and micturition problems after surgery compared with women, whereas women experienced more appetite loss [86]. There were differences between males and females in relation to the symptoms that significantly correlated with HRQoL over time (Table 1) [86].

**Table 1:**
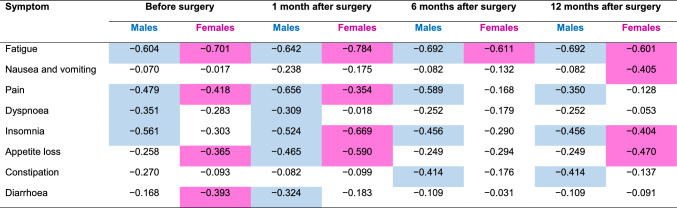
Correlations (*R* values) between global health status and symptom scales in male and female patients after sphincter-saving surgery for rectal cancer in Japan

Shaded cells indicate parameters with a significant correlation [86]. Adapted from Table 4 in [86], by arranging time in columns (instead of rows), including only symptoms (not functioning), and shading those cells with significant differences, under a CC BY 4.0 DEED license (https://creativecommons.org/licenses/by/4.0/)

The severity of haematological and acute organ toxicity associated with chemoradiotherapy is significantly worse in female than male patients who receive these treatments in the neoadjuvant or adjuvant setting [83, 87]. According to one analysis, the effect was particularly marked for intestinal toxicity, but bladder toxicity was similar in male and female patients [87]. Recovery of bowel and sexual function are also affected by preoperative radiotherapy. Preoperative irradiation slows recovery of bowel and sexual function after total mesorectal excision and negatively affects sexual function in both men and women [88]. The impact can persist for many years; even 14 years after preoperative radiotherapy, HRQoL remained lower than in the general population, and both men and women continued to experience sexual dysfunction after irradiation [89].

### Toxicity of systemic treatments

Women receiving systemic therapy for cancer generally experience a higher incidence of symptomatic AEs, irrespective of the type of treatment (chemotherapy, immunotherapy or targeted therapy) [90]. In patients with CRC, there is a consistently higher incidence and severity of haematological and non-haematological AEs amongst female than male patients receiving fluoropyrimidine-based adjuvant chemotherapy regimens [71, 81, 91, 92], likely as a result of greater systemic exposure in women due to pharmacokinetic factors (described earlier). Females are also more likely than males to discontinue fluoropyrimidine-based chemotherapy as a result of AEs [81].

The increased risk of chemotherapy-related AEs amongst females is also seen in the metastatic setting [73, 93]. In the PanaMa study, the incidence of nausea and alopecia of any grade was significantly higher in female than male patients who had been randomised to treatment with panitumumab + chemotherapy [81].

On the other hand, data from Canada indicated that male sex were a risk factor for developing any thromboembolic event (OR 1.20; 95% CI 1.08–1.34) and coronary thromboembolism (OR 2.15; 95% CI 1.74–2.64) in patients with CRC [94]. This study included any thromboembolic event, not just those occurring during systemic treatment, but noted that treatment with bevacizumab or fluoropyrimidines also significantly increased the risk of any thromboembolism and venous thromboembolism [94]. However, the relationship between male sex and thromboembolism was no longer significant when the analysis was limited to patients receiving fluoropyrimidine-based chemotherapy (OR 1.16; 95% CI 0.98–1.37) [94].

Men also appear to be at increased risk of developing hypersensitivity reactions to oxaliplatin, according to a 5-year real-world analysis from Thailand [95]. In this study, male sex was associated with a significantly increased risk of oxaliplatin hypersensitivity (OR 2.628 l 95% CI 1.450–4.763; p = 0.001), after adjustment for other risk factors [95].

In the metastatic setting, the timing of irinotecan administration may be relevant to AE occurrence, particularly in female patients. Data show that, in females, the incidence of grade 3 or 4 AEs is lower when irinotecan is administered in the afternoon compared with the morning or at night, whereas in males, toxicity is minimised by morning administration (vs afternoon or night) [96].

Not all of the differences in chemotherapy-related toxicity between men and women may result from inherent biological differences; some may be gender-related. Gender stereotypes, stigma and social norms influence both the health-seeking behaviour of the affected individuals and the attitudes of healthcare practitioners towards the patient [97]. For example, men may be less willing to report or discuss side effects with their physician due to gender norms about being ‘manly’ and ‘stoic’ [98]. In addition, gender may influence whether or not a patient sees a side effect as ‘adverse’ or the level of distress a patient feels about a particular side effect [97]. Women are particularly vulnerable to body image distress [99, 100], which affects how they feel about side effects such as weight gain or alopecia [97]. They are also more likely than men to experience psychological distress (including anxiety and depression) which may also affect their experience of AEs such as bowel symptoms, and vice versa (i.e. adverse bowel or urinary symptoms may worsen psychological distress) [99]. All of these factors can affect AE reporting in clinical trials and in clinical practise.

## Recommendations and future lines of action

Given the sex-related differences in CRC described above, we recommend a number of measures that can be implemented in Spain and elsewhere to improve our knowledge base, and hopefully lead to better outcomes for male and female patients.

First, we need to consistently apply sex- and gender-related terms in medical records and clinical research, and record both sex and gender in patient registries and clinical databases. This is necessary so that research can identify both sex- and gender-related issues in the delivery and effects of cancer services, such as cross-sectional studies to identify potential sex and gender bias in access to services. Other areas of research include the need for sex-specific thresholds for faecal haemoglobin during screening.

Second, a greater number of longitudinal studies are needed to examine the impact of sex and gender on outcomes. This includes more research on the impact of gender norms on health-related behaviours in CRC, such as screening participation or AE reporting. Potential research may include cohort studies examining the impact of sex and gender on treatment outcomes, including toxicity, and on patients’ experience of treatment. Another important research imperative is to identify the most appropriate body composition parameters to guide chemotherapy dosing, e.g. bodyweight, body surface area or fat-free mass.

Third, we need to raise awareness amongst clinicians of the potential for sex and gender bias in treatment and dosing decisions, through educational initiatives at a national, regional and local level. Such educational initiatives would be strengthened by data from the type of research described above. Only when physicians truly understand how CRC affects men and women differently can they individualise treatment decisions for each patient.

## Conclusions

There are clear differences between men and women in the epidemiology, presentation and outcomes of CRC, some of which may be sex-related (due to inherent biological mechanisms) and some of which may be gender-related (due to lifestyles, access or experiences). Further research is needed to clarify these differences, so that treatment can be individualised for a patient’s own needs.

## Data Availability

Data sharing is not applicable to this article as no datasets were generated or analysed.
